# In regard to Cuccia et al.: impact of hydrogel peri-rectal spacer insertion on prostate gland intra-fraction motion during 1.5 T MR-guided stereotactic body radiotherapy

**DOI:** 10.1186/s13014-020-01642-z

**Published:** 2020-08-17

**Authors:** Hamed Ghaffari, Mahmoud Navaser, Soheila Refahi

**Affiliations:** 1 Department of Medical Physics, School of Medicine, Iran University of Medical Sciences, Tehran, Iran; 2 Department of Medical Physics, Faculty of Medicine, Ardabil University of Medical Sciences, Ardabil, Iran

**Keywords:** Prostate cancer, Prostate motion, Hydrogel spacer, SBRT

## Abstract

We read the article entitled “Impact of hydrogel peri-rectal spacer insertion on prostate gland intra-fraction motion during 1.5 T MR-guided stereotactic body radiotherapy” with great interest. In that study, the author reported that there is a statistically significant difference in the rotational antero-posterior shifts between the spacer and the non-spacer groups. Also, there was no statistically significant difference between the groups in terms of translational shifts. However, there are some points about the study. In this letter, we aimed to clarify these points.

To the Editor:

We read with great interest the Cuccia et al. study investigating the effect of hydrogel rectal spacer on prostate intra-fraction motion during 1.5 T magnetic resonance imaging (MRI)-guided stereotactic body radiotherapy (SBRT) [1]. In the study, they reported that there is a statistically significant difference in the rotational antero-posterior shifts between the spacer (*n* = 10) and the non-spacer (n = 10) groups (*P = 0.033*), with respective median values of − 0.0005° and 0.09°. Also, the authors observed no statistically significant difference between the groups in terms of translational shifts. We appreciate the authors’ efforts to elucidate the role of peri-rectal hydrogel spacer on prostate motion during MR-guided prostate SBRT, as primary endpoint. However, there are some points about the study that need to be clarified.

First, the results of recent systematic review show that although many studies have reported that rectal hydrogel spacer insertion significantly reduces rectal wall doses and radiation-induced rectal toxicities, resulting in improving patient quality of life during prostate radiation therapy, the application of prei-rectal hydrogel spacer does not reduce inter- and intra-fractional prostate motion [2]. It is worthwhile to mention that the insertion of hydrogel spacer increase the perirectal space, when it is injected between Denonvilliers’ fascia and the anterior rectal wall, thereby reducing rectal radiation doses and rectal toxicities [3, 4]. Of note, hydrogel spacer is not really able to fix the pelvic organs such as prostate because it cannot control rectal filling. On the contrary, several studies have demonstrated that the use of Endorectal balloons and rectal retractor can significantly reduce intra-fractional prostate motion because these devices mainly aim to fix the rectal volume/position and consecutively the prostate motion and by doing that, expanding the rectal wall and therefore sparing more portions of the wall by using a smaller planning target volume (PTV) [2, 5, 6]. One can hypothesize, that the application of hydrogel spacer may reduce prostate motion by squeezing the prostate gland towards the pubic bone, as the possible mechanism. Conversely, the injection of hydrogel spacer may increase pressure on the rectal wall causing rectal irritation and discomfort, resulting in more prostate motion [2, 7].

Second, the authors evaluated intra-fractional prostate displacements by means of quantifying pre- and post-fractional deviations of the prostate by MRI. Using MR imaging is considered as an ideal scenario for image-guided prostate radiotherapy because of high soft tissue contrast [8]. It should be noted, however, that using pre- and post-treatment MRI cannot well clarify the impact of hydrogel spacer on intra-fractional prostate motion. In other words, real intra-fractional prostate shifts cannot be accurately mapped by measuring the position of the prostate immediately before and after a radiotherapy treatment fraction. Furthermore, the authors did not use Cine-MRI to track prostate intra-fraction motion over the full beam-on period. Real-time motion tracking can clearly elucidate the effect of hydrogel spacer on prostate intra-fraction motion, as compared to pre-post imaging.

Third, an interesting point of the study is that the authors assessed both intra-fraction translational and rotational shifts. The authors considered no rotational and translational shift tolerance. We think that a shift tolerance value could be defined and the shifts within tolerance value and greater than it between the spacer and non-spacer cohorts could be compared.

Fourth, the aim of managing prostate motion is rectal toxicity reduction and increased efficacy by a lower target miss rate; therefore, it is necessary to assess the impact of rectal displacement devices (i.e.*,* Endorectal balloons, hydrogel spacer, and rectal retractor) on target margins and oncological outcomes during prostate radiotherapy. There have been numerous studies to assess the effect of rectal displacement devices on prostate motion [2]; however, the relationship between rectal displacement devices, target margins, and toxicity data has yet to be clarified. In fact, motion and toxicity data should be reported together. Therefore, although the study has not evaluated the effect of hydrogel spacer on target margins, it is the first study reporting motion and toxicity data when a hydrogel is used during prostate radiotherapy.

In summary, peri-rectal hydrogel spacer may serve as an immobilization device for prostate, but further studies with a large sample size will be required to evaluate this hypothesis.

## Data Availability

No data generated.

## Abbreviations

MRI: Magnetic resonance imaging
SBRT: Stereotactic body radiotherapy
PTV: Planning target volume
